# Evidence of Rabies Virus Exposure among Humans in the Peruvian Amazon

**DOI:** 10.4269/ajtmh.2012.11-0689

**Published:** 2012-08-01

**Authors:** Amy T. Gilbert, Brett W. Petersen, Sergio Recuenco, Michael Niezgoda, Jorge Gómez, V. Alberto Laguna-Torres, Charles Rupprecht

**Affiliations:** National Center for Emerging and Zoonotic Infectious Diseases, Centers for Disease Control and Prevention, Atlanta, Georgia; Dirección General de Epidemiología, Ministerio de Salud, Lima, Perú; Epidemic Intelligence Service, Centers for Disease Control and Prevention, Atlanta, Georgia; Virology Department, US Naval Medical Research Unit 6, Lima, Perú

## Abstract

In May of 2010, two communities (Truenococha and Santa Marta) reported to be at risk of vampire bat depredation were surveyed in the Province Datem del Marañón in the Loreto Department of Perú. Risk factors for bat exposure included age less than or equal to 25 years and owning animals that had been bitten by bats. Rabies virus neutralizing antibodies (rVNAs) were detected in 11% (7 of 63) of human sera tested. Rabies virus ribonucleoprotein (RNP) immunoglobulin G (IgG) antibodies were detected in the sera of three individuals, two of whom were also seropositive for rVNA. Rabies virus RNP IgM antibodies were detected in one respondent with no evidence of rVNA or RNP IgG antibodies. Because one respondent with positive rVNA results reported prior vaccination and 86% (six of seven) of rVNA-positive respondents reported being bitten by bats, these data suggest nonfatal exposure of persons to rabies virus, which is likely associated with vampire bat depredation.

## Introduction

Rabies is caused by single-stranded negative-sense RNA viruses in the genus *Lyssavirus*. Rabies virus (RABV; genotype I) is the most prolific of the 12 viral species classified within the genus, and it is responsible for greater than 55,000 human deaths annually.^1^ Typically, RABV is transmitted in the saliva after the bite of an infected mammal. In the Americas, bats and carnivores are the major reservoirs of RABV.^2^ Multiple insectivorous bat species play a role in RABV transmission to humans in the United States.^3^ In Latin America, RABV is transmitted principally by the common vampire bat (*Desmodus rotundus*), although several other Neotropical bat species play a role in RABV circulation.^4^–^8^ Rabies is the most recognized human health risk from bats in Latin America, with dual impacts for public health and agriculture.^9^ Wide circulation of RABV among vampire bats throughout their geographic range is shown by extensive reports of vampire bat-associated RABV infections in bats, humans, and cattle throughout Latin America.^8^,^10^–^12^

In Perú, outbreaks of rabies linked to vampire bat bites have been documented among populations living in the Amazon region over the past several decades.^4^,^12^–^14^ Approximately 81% (113 of 139) of the human rabies cases reported in Perú from 1996 to 2010 were associated with vampire bats.^12^,^15^ In April of 1996, an outbreak of rabies resulted in at least nine human deaths in two Amazonian villages. Samples from the victims were characterized as RABV associated with *D. rotundus*.^13^ Between December of 2006 and February of 2007, an outbreak involving 527 persons bitten by vampire bats claimed at least 23 deaths in southeastern Perú, all implicating *D. rotundus*.^4^ In 2009, 19 cases of human rabies transmitted by vampire bats were reported from five outbreaks located in the Amazon region.^16^–^18^ From December of 2009 to February of 2011 in the District of Nieva, 14 suspected human rabies deaths were reported, and two of the cases were confirmed by direct fluorescent antibody testing.^19^ Most recently, from February to July of 2011, at least 20 suspected human rabies cases (18 children and 2 adults) among indigenous persons from the Aguaruna tribe in the District of Imaza were reported.^20^ Common risk factors for human RABV infection in the Amazon include poor housing conditions, small population groups in remote areas, poor access to health services, and a general lack of awareness or cultural barriers regarding the transmission of rabies by bats. There is ample evidence of frequent depredation by vampire bats on humans and livestock in the Peruvian Amazon but inadequate investigation of human response to RABV exposures among persons at risk living in this region.

Rabies has the highest case fatality rate of any conventional infectious disease, approaching 100%. The likelihood of a productive rabies infection after exposure to a lyssavirus is known to depend on a variety of factors, including but not limited to dose, route of exposure, site of exposure, variant, host genetic makeup, pre- and/or post-exposure prophylaxis (PreEP and PEP, respectively), etc.^21^ All mammals are susceptible to lyssavirus infection, but species-level variation in susceptibility has long been recognized.^2^ Among reservoir species, foxes and other canids seem to be quite susceptible to RABV infection, characterized by little to no (e.g., 0–5%) rabies virus neutralizing antibody (rVNA) seroprevalence (i.e., animals developing virus neutralizing antibodies after RABV exposure) among natural populations.^22^,^23^ Contrastingly, bat populations seem to be less susceptible to RABV infection and are characterized by relatively high (e.g., 5–50%) rVNA seroprevalence in the wild.^24^–^26^ One experiment suggested that non-human primates might be resistant to infection from rabid bats,^27^ but bat-associated human rabies deaths worldwide show human susceptibility to lyssavirus infection by bat bite.^3^,^28^–^30^ The work by Bell^31^ argued provocatively that abortive RABV infections were readily observed and reproducible in animals, and thus, they should be considered a possible outcome for humans. Reports of human survival after rabies infection, despite clinical presentation, are quite rare in the literature.^32^–^37^ However, a recent case of presumed abortive rabies infection in the United States highlights a rare event of human survival after presentation of clinical symptoms and minimal intervention treatment.^38^

The objective of this study was to investigate risk factors for bat and RABV exposure in Amazonian communities that were suspected to be at high risk of vampire bat depredation based on their proximity to recent outbreaks in Perú and rural living conditions. A survey questionnaire was used to capture demographic information of the study populations, salient details of any previous bat exposure, and self-reported vaccination history among respondents sampled.

## Materials and Methods

### Sample collection.

**Figure 1.**
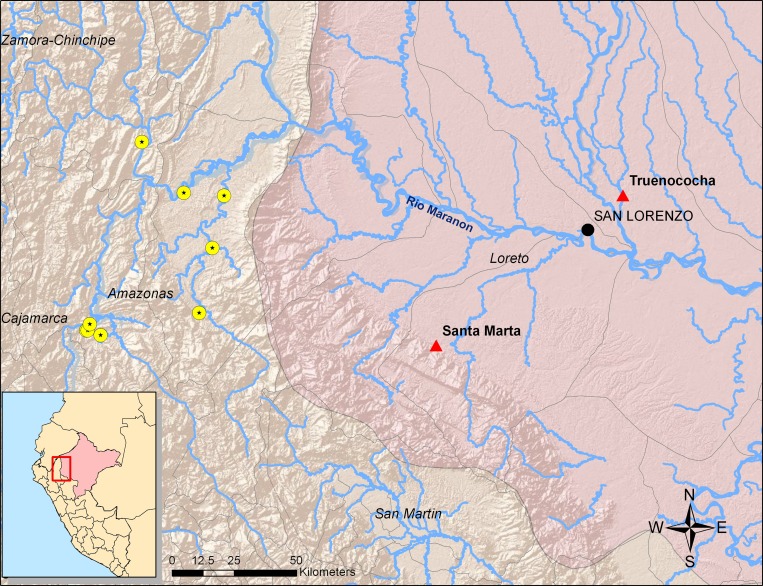
Map of the study location with the triangles indicating the communities surveyed and the circles with stars indicating the locations of previous human rabies outbreaks associated with to vampire bats.

Two communities were surveyed in May of 2010 in the Province Datem del Marañón in the Loreto Department of Perú (Figure 1). Samples were collected as part of a survey to evaluate bat–human interactions and rabies risk in the Amazon. The survey protocol was approved by the Centers for Diseases Control and Prevention (United States) and the Hospital Nacional Dos de Mayo (Perú) Institutional Review Boards in compliance with all applicable federal regulations governing the protection of human subjects. Both communities could only access the nearest health post in the town of San Lorenzo by river. The community of Truenococha is in the District of Pastaza, had an estimated population of 111, is of mestizo (i.e., mixed) cultural descent, is approximately 2 hours from the nearest health post by motorized boat (∼50 km), and was raising approximately 12 head of cattle at the time of the study. The community of Santa Marta is in the District of Cahuapanas, had an estimated population of 205, is of indigenous cultural descent, is located approximately 6–8 hours from the nearest health post by motorized boat (∼85 km), and was not raising any cattle at the time of the study. Residents from both communities were enrolled in a knowledge, attitudes, and practices (KAP) survey about prior bat exposure, health-seeking behaviors, knowledge of rabies, and prior rabies PreEP or PEP. A 2- to 5-mL blood sample was collected from consenting respondents by an aseptic technique. Sera were separated by centrifugation, stored in liquid nitrogen until transfer to ×80°C, and shipped from Perú to the Centers for Disease Control and Prevention in Atlanta, Georgia.

### rVNA.

Sera were screened for rVNA by the rapid fluorescent focus inhibition test (RFFIT) as described.^39^ Briefly, sera were screened individually at a 1:5 and 1:25 dilution against a constant dose of rabies virus (50 focus forming doses [FFD_50_]). Sera that exhibited complete neutralization of RABV at a 1:5 dilution were considered rVNA positive using the recommendations of the Advisory Committee on Immunization Practices (ACIP).^21^ Sera that were rVNA positive were screened at additional dilutions up to 1:390,625 to determine endpoint titers using the Reed–Muench method.^40^ These data were converted to international units per milliliter^−1^ by comparison with the US Standard Rabies Immune Globulin (SRIG; Laboratory of Standards and Testing, Food and Drug Administration, USA) diluted to 2 IU mL^−1^.

### Indirect fluorescent antibody assay.

Sera were screened for RABV ribonucleoprotein (RNP) immunoglobulin M (IgM) and IgG antibodies by the indirect fluorescent antibody (IFA) test as described^41^ using affinity-purified, fluorescein-labeled goat anti-human antibodies (Kirkegaard & Perry Laboratories, Inc., Gaithersburg, MD). Sera were screened individually at dilutions of 1:4 to 1:128 with IgM or IgG preparations, respectively. A positive reaction at any of these dilutions was considered evidence of RABV-specific IgM or IgG antibodies.

### Statistical analyses.

Separate analyses were conducted with survey data. The first analysis stratified all respondent data by exposure history to bats (i.e., with exposure defined as a bat bite or scratch or touching a bat with unprotected skin) to evaluate risk factors for bat exposure. The second analysis focused only on individuals from whom a serum sample was obtained and tested, with stratification of respondent data regarding bat exposure by serological status to evaluate risk factors for rabies virus exposure. All statistical analyses were performed using SAS v.9.3 (SAS Institute, Cary, NC). Fisher exact test was used to evaluate associations (α = 0.05) between the response (stratification) variable (i.e., bat exposure or serological status) and factors such as community of residence, age, sex, education level, and self-reported bat exposure history, which includes subcategories of bite, scratch, and/or touching a bat with unprotected skin.

## Results

A total of 92 persons were interviewed from 51 households and represented a total community population of 316 persons (Table 1). The mean age of all respondents was 25 years (range = 2–67 years), and 55% (51 of 92) of respondents were male. Among respondents, 82% (62 of 76) of persons interviewed reported completing a primary education or less. Among households, 86% (44 of 51) owned pets or livestock, and 61% (27 of 44) of households owning pets or livestock reported that their animals were bitten by bats. Among the total community population, 23% (73 of 316) of persons had exposure to bats. Although a biased sample, among persons interviewed, 54% (50 of 92) reported being bitten by bats previously.

Among the surveyed populations, several risk factors for exposure to bats were identified (Table 2). The most significant risk factor for exposure to bats was community of residence, with Santa Marta having a greater proportion of exposed persons (odds ratio [OR] = 8.71, *P* < 0.001). Other significant factors included age, with persons aged 25 years or younger having a greater risk of bat exposure (OR = 3.59, *P* = 0.038), and households reporting pets or livestock bitten by bats (OR = 8.83, *P* < 0.001). Furthermore, households with more than five family members (OR = 3.41, *P* = 0.03) were at higher risk of bat exposure. Contrastingly, persons who reported living at their house for less than 1 year were at a lower risk for bat exposure compared with other respondents (OR = 0.20, *P* = 0.02).

Among 63 sera obtained from individual respondents (age range = 2–62 years, mean = 29 years; overall male to female ratio is 1.85), 11% (7 of 63) showed an rVNA titer (range = 0.1–2.8 IU mL^−1^). From the IFA test, RABV RNP IgM and IgG antibodies were detected from 4 of 63 samples (Table 3). The rVNA seroprevalence was lower in Truenococha (5%; 1 of 19) compared with Santa Marta (14%; 6 of 44), contrasting the trend in IFA antibody seroprevalence in Truenococha (11%; 2 of 19) and Santa Marta (5%; 2 of 44). Among seropositive respondents from either community, all (9 of 9) reported bat exposure, which was defined as a bat bite, scratch, or direct contact with unprotected skin. Furthermore, 75% (6 of 8) of unvaccinated seropositive respondents reported a history of a bat bite (Table 3). Only one seropositive respondent reported having received rabies PEP, although vaccination history details could not be elicited from two other seropositive respondents.

Seropositive status of an individual was associated with age, with persons aged 29 years or less being at significantly lower risk of being seropositive (OR = 0.08, *P* = 0.01) (Table 4). Seropositive status was not associated with community of residence, gender, or education level. Although a greater proportion of seropositive persons reported bat exposure (9 of 9; 100%), including bat bite (7 of 9; 78%) or touching a bat (5 of 9; 56%), the differences were not significant compared with the reported bat esposure of seronegative persons (36 of 48; 75%), including bat bite (31 of 48; 65%) or touching a bat (16 of 48; 33%) (Table 4).

## Discussion

Despite a wealth of studies documenting natural seroprevalence among wildlife reservoirs, few prior studies have reported natural human seroprevalence to RABV. One study showed rVNA among 7% (2 of 30) of sera from raccoon hunters in Florida, although at low titers (∼0.1 IU mL^−1^).^42^ Another study, among Canadian Inuit hunters having animal contact but no vaccination history for RABV, also detected rVNA in 29% (9 of 31) of individuals.^43^ However, titers in that study were also uniformly low (< 0.1 IU mL^−1^). A later study among fox trappers in Alaska reported rVNA among 12% (3 of 26) of individuals.^44^ Two of three seropositive trappers had a previous vaccination history. The single seropositive Alaska fox trapper who had not received rabies vaccine previously had a high rVNA titer (2.3 IU mL^−1^), perhaps associated with a 47-year history of trapping and skinning foxes (without personal protective equipment) and a cumulative harvest of over 3,000 foxes. During a human rabies outbreak investigation in the Department of Amazonas in Perú in 1990, 17% (8 of 48) of persons in two affected communities were seropositive for rVNA, one of whom later died.^14^ In the study by Lopez and others,^14^ the median rVNA titer among the seven surviving persons was 0.18 IU mL^−1^ (range = 0.14–0.66 IU mL^−1^), whereas the person who died had a titer of 7.6 IU mL^−1^ at the time of sampling. Because the study by Lopez and others^14^ did not detect a statistical relationship relating to age of the individuals or exposure to bats with antibody concentration, all of the positive rVNA titers among the seven survivors were considered to be nonspecific. Despite potential for low-titer, false-positive neutralizing antibody titers resulting from nonspecific inhibition of virus growth, a recent review did not suggest evidence of nonspecific inhibition of virus growth at serum dilutions of 1:25 or greater in serological neutralization assays, although they were based on observations among non-indigenous persons.^45^ In the current study, a 50% reduction of fluorescing fields at a 1:25 serum dilution would have resulted in a titer of 0.2 IU mL^−1^, and given that six of seven rVNA titers were greater than 0.2 IU mL^−1^, these data do not suggest a high potential for nonspecific inhibition. The single respondent with an rVNA titer below 0.2 IU mL^−1^ (and RNP IgG titer of 1:8) in this study also reported a history of vaccination (Table 1), which is a more parsimonious explanation for her seropositive status. It is also noteworthy that none of the respondents in either community (seropositive or otherwise) reported the preparation or consumption of bats as a food source.

The observation of unvaccinated seropositive respondents, in the context of a self-reported history of bat bites in an area endemic for vampire bat rabies, suggests that RABV exposure is not invariably fatal in humans. A genetic basis for susceptibility and immunological response to rabies has been shown previously in mice.^46^–^48^ Although it is possible that certain isolated and remote populations in the Amazon region may be genetically and immunologically unique,^49^,^50^ two studies have also found signatures of gender-specific genetic admixture in certain Amazon populations and suggest that historical social policies have strongly influenced the migration of persons to and genetic mixing within the Amazon region.^49^,^51^ Genetic comparisons of immunological markers and relevant inducible responses (e.g., humoral and cellular response to rabies vaccination)^52^ from populations in urban areas and throughout the Amazon region of Perú may shed additional light on whether certain indigenous populations show evidence of natural selection for enhanced nonspecific or specific immunological responses and genetic resistance to rabies infection.

Individual immune response to natural infection with RABV may include virus-specific binding and neutralizing antibodies depending on factors such as viral dose, degree of replication in the periphery, and successful entry and replication in the central nervous system (CNS). Both RABV antibodies to the glycoprotein and RNP have a proven role in the immune response after vaccination.^53^,^54^ Based on patient histories in the United States, RABV RNP binding antibodies are typically detected first by IFA in response to clinical infection, and rVNA may or may not be induced.^38^ These observations suggest an early response of antibodies to RNP relative to the induction of rVNA during CNS infection. Based on the degree of peripheral replication, there may be infected cells and budding of intact virions or apoptosis of infected cells presenting RNP. Although the data presented in this study cannot conclusively differentiate between scenarios of abortive peripheral viral infection or clearance of a small viral dose insufficient to establish infection, the seropositive responses show exposure to RABV in the absence of vaccination.

The presence of rVNA in unvaccinated subjects implies prior viral exposure but not necessarily viral replication, which can be shown by the induction of rVNA responses to even a single dose of inactivated rabies vaccine.^55^ However, given that rabies vaccination is accomplished with large doses of purified inactivated RABV virions, it remains unclear whether replication is a prerequisite for induction of humoral or cellular responses to natural exposures involving smaller doses of street RABV. In an experimental infection of bats with varying doses of RABV, low-dose RABV exposures did not lead to productive CNS infection, and apparently, they were cleared by an immune response in the periphery.^56^ Previous studies have shown that RABV-specific antibodies are not uniformly induced in the serum or cerebrospinal fluid (CSF) of clinical human rabies cases who do not receive rabies vaccine or immune globulin treatment, with greater probabilities of serological detection in patients with longer morbidity periods (i.e., days alive after onset of clinical symptoms).^57^–^59^ This report identifies a higher risk for bat exposure among young persons, despite finding a greater risk of rabies virus exposure (i.e., seropositive status) among older persons. It is plausible that multiple low-dose RABV exposures are needed to induce the rVNA responses observed in this study, consistent with the observed correlation of seropositive status with age. Evidence of RABV-specific antibodies in serum and CSF of subjects who did not receive rabies vaccine or immune globulin has been interpreted as evidence of viral replication and an abortive infection.^33^,^38^ The data in this study are inconclusive with regard to abortive infection in the seropositive respondents, because CSF samples were not collected, thus precluding evidence of RABV invasion into the CNS. Responses to interview questions about prior or current illness (and associated symptoms) did not support a history of CNS infection among respondents in this study.

Innate immunity is typically another important component in combating viral infections. Prior studies have suggested that street RABVs tend to evade induction of the host innate immune response and particularly, interferon and inflammatory pathways.^60^ This finding is consistent with the observations of an inverse relationship between RABV virulence and the degree of viral replication, with highly pathogenic RABVs showing limited levels of replication, G protein accumulation, and apoptotic signaling in infected neurons.^61^,^62^ Although one study showed that a bat (street) RABV replicates efficiently in nonneuronal cells,^63^ it has been suggested that limited replication in the periphery may be an adaptation of highly neuroinvasive street RABVs to minimize a peripheral host immune response *in vivo*, thus facilitating entry into the CNS.^61^ Minimal immune induction in the periphery may be expected under scenarios of successful street RABV infection (i.e., CNS invasion); however, none of the respondents reported symptoms suggestive of CNS involvement.

Rather than invoking peripheral viral replication as a requisite to the induction of rabies-specific serum antibody, one could also consider a dirty bite hypothesis. Little is known about (1) the population of RABV particles transmitted in the saliva during an animal bite and (2) what other substances or organisms may also be present. It is unrealistic to assume that homogenous populations of completely intact RABV virions are passed in the saliva, particularly given reports of defective interference (DI) particles.^64^ Furthermore, it cannot be ruled out that there are other properties or normal flora organisms associated with saliva from an animal bite that contribute to induction of a nonspecific innate and inflammatory immune response to the wound in the absence of peripheral RABV replication. These data highlight important complexities concerning the interpretation of serology, which is currently the only diagnostic tool that has been successful in antemortem diagnosis of nonfatal cases of human rabies infection.

Prior vaccination history could confound the interpretation of the serological data in this study. Human rabies cell culture and nerve tissue vaccines are inactivated and do not replicate in recipients,^65^ but they induce robust rVNA responses.^66^,^67^ Equivocal evidence has been published regarding induction of non-neutralizing RNP antibody after rabies vaccination.^59^,^68^,^69^ It is notable that suckling mouse brain vaccine (SMB) is used in Perú for rabies PreEP and PEP, although PreEP is typically restricted to persons at occupational risk of infection. Only one seropositive respondent reported receiving rabies PEP. Data were unclear regarding self-reported prophylaxis among two other respondents. Given the remote location of these villages, our collaboration with personnel from the nearest health post that would have administered PEP during an intervention and the vaccination history reporting among other respondents, it is unlikely that the other eight seropositive respondents received rabies PreEP or PEP. Furthermore, persons living in this region often do not understand the real significance of being bitten by a vampire bat and are unlikely to seek medical assistance after a bite or may actively avoid modern medical care because of traditional beliefs.^70^

Prior reports of human rabies outbreaks in the Amazon, including 11 cases in the Department of Loreto in Perú in 1995, the results of this study, and nearby recent vampire bat-associated outbreaks suggest a high rabies risk in the Peruvian Amazon (Figure 1).^12^,^15^ Vampire bats principally feed on cattle or other mammals (including humans) when livestock is not widely available.^71^,^72^ Seasonal incidence of RABV infections from vampire bats to humans and cattle occurs purportedly shortly after the onset of the rainy season.^14^,^73^,^74^ However, reports of outbreaks during the dry season have also exist.^75^,^76^ Regardless of season, several reports indicate stronger coincidence of vampire bat depredation on humans after the elimination of livestock, such as pigs or cattle.^14^,^74^,^77^ In the current study, despite the observation that nearly equal proportions of exposed and non-exposed respondents reported owning pets or livestock (Table 2), exposed persons were more likely to report that their pets or livestock had been bitten by bats, presumably with greater risk when the bitten animals are confined close to one's residence. Interestingly, Santa Marta respondents exhibited nearly a ninefold greater risk for bat exposure, which may be non-exclusively influenced by other identified risk factors in the survey, including a greater proportion of younger persons and greater proportion of pets or livestock bitten in Santa Marta (Table 1). However, other factors not captured in the survey may also contribute to this observation, such as the absence of cattle in Santa Marta, asymmetry in the proximity of bat roosts to these communities, or some other unique ecological feature, although it is relevant to note that a greater proportion of Truenococha respondents reported entering a bat refuge (Table 1). Greater household size also contributed to increased risk for bat exposure, and it is possible that larger families have a greater proportion of young children, leading to greater risk of bat exposure. It is clear that there are a variety of factors that can influence individual and household risk of bat exposure in this region. Greater replication and geographic representation of communities in the Amazon would identify the most robust factors that contribute to geospatial variation in risk for bat and RABV exposure.

Through evidence presented in this study and one earlier report,^14^ it is evident that a substantial fraction of the human population living in remote areas of the Peruvian Amazon experiences regular depredation by vampire bats and likely exposure to RABV. Seasonal patterns of vampire bat depredation in this region of the Amazon have not been well-characterized, and the majority of persons interviewed did not identify any apparent seasonality. Regardless, it is plausible that some individuals experience nonfatal exposure to RABV by vampire bat bites, with subsequent exposures leading to an immunological boost or anamnestic response. Regardless, all persons living in these communities should be considered for rabies prophylaxis as part of any subsequent intervention. New paradigms, such as rabies PreEP for Amazon populations at risk, may be necessary to prevent and control rabies in such unique ecological circumstances.

In closing, it is relevant to recognize that the number of newly discovered lyssaviruses has increased significantly in recent decades. Pre-1980, traditional nomenclature recognized just four *Lyssavirus* genotypes (i.e., RABV, Lagos bat virus [LBV], Duvenhage virus [DUVV], and Mokola virus [MOKV]), whereas there are now 12 recognized species within the genus, 11 of which are presumed to have bats as the primary reservoir host.^78^ Although earlier studies questioned the pathogenicity of certain subsets of lyssaviruses, namely the phylogroup 2 viruses (e.g., LBV and MOKV),^79^ experimental studies have shown that phylogroups 1 and 2 lyssaviruses are pathogenic in animal models, including bats,^80^–^83^ and human infections with phylogroups 1 and 2 lyssaviruses are reviewed in the work by Banyard and others.^84^ The absence of human infections linked to certain lyssaviruses need not be misinterpreted as variation in pathogenicity of those lyssaviruses for three reasons: (1) a near absence of any systematic surveillance system for reporting and detecting human rabies infections in many parts of the world where these viruses are endemic,^85^ (2) the overwhelming burden of canine-associated human RABV infections in many of these same places (i.e., throughout Africa and Central and Southeast Asia), which could obscure less frequent bat-associated infections,^1^ and (3) the excellent sensitivity of the current gold standard fluorescent antibody test to detect any lyssavirus infection but inability of this test to type the specific lyssavirus implicated in infection. Although it is likely that there are undiscovered lyssaviruses in the Old World (figure 1b in the work by Rupprecht and others^78^), RABV is the only lyssavirus known to be present in the New World. After the advent and transfer of technologies such as monoclonal antibody typing and nucleic acid detection and sequencing methods throughout the Americas and along with regional campaigns for canine rabies elimination in the Americas and increased characterization of human rabies infections to achieve this goal, there still have not been any new lyssaviruses discovered in the Americas.^5^,^11^,^86^,^87^ For these reasons, a hypothesis that the results in this study reflect cross-reactivity to an undiscovered lyssavirus is one for which evidence is lacking but is also not an explanation that can be ruled out. However, hypotheses that there are undiscovered and non-pathogenic lyssaviruses that could be implicated as a mechanism for non-lethal rabies exposure showed in this study seem unsubstantiated based on evidence of lyssavirus pathogenicity in humans and animals worldwide.

## Supplementary Material

Supplemental Questionnaire.

## Figures and Tables

**Table 1:** Demographic and population characteristics of the two communities surveyed in the Province Datem del Marañón of Perú, 2010

Category	Santa Marta	Truenococha	Total
Households visited	29	25	54
Households enrolled (percent of total visited)	28 (96.6%)	23 (92.0%)	51 (94.4%)
Individuals interviewed	68	24	92
Estimated community population (sum of persons reported living in each household)	205	111	316
Mean age in years (range)	21.3 (2–49)	35.6 (5–67)	25.0 (2–67)
Median age in years (range)	21.5 (2–49)	30.5 (5–67)	25.5 (2–67)
Male (percent of total interviewed)	35 (51.5%)	16 (66.7%)	51 (55.4%)
Education (*N* = 76)			
Primary or below	44 (84.6%)	18 (75.0%)	62 (81.6%)
Secondary or above	8 (15.4%)	6 (25.0%)	14 (18.4%)
Households with pets or livestock (percent of households)	26 (92.9%)	18 (78.3%)	44 (86.3%)
Households with pets or livestock bitten by bats (percent of households with pets or livestock)	19 (73.1%)	8 (44.4%)	27 (61.4%)
Households with one or more bat exposures* (percent of households)	25 (89.2%)	11 (47.8%)	36 (70.6%)
Individuals with one or more bat exposures* (percent of estimated community population)	61 (29.8%)	12 (10.8%)	73 (23.1%)
Bat bite (percent of total interviewed)	44 (64.7%)	6 (25.0%)	50 (54.3%)
Bat bite more than one time per year	18 (26.5%)	0	18 (19.6%)
Bat bite within the last 6 months	31 (45.6%)	1 (4.2%)	32 (34.8%)
Bat contact with unprotected skin (percent of total interviewed)	16 (23.5%)	9 (37.5%)	25 (27.2%)
Skin contact more than one time per year	3 (4.4%)	4 (16.7%)	7 (7.6%)
Skin contact within the last 6 months	10 (14.7%)	5 (20.8%)	15 (16.3%)
Bat scratch (percent of total interviewed)	2 (2.9%)	1 (4.2%)	3 (3.3%)
Individuals reporting entering a bat cave or refuge (percent of total interviewed)	5 (7.4%)	6 (25.0%)	11 (12.0%)
Individuals reporting eating or cooking a bat as food (percent of total interviewed)	0	0	0
Rabies serology (*N* = 63)			
RVNA positive (percent total tested)	6 (13.6%)	1 (5.3%)	7 (11.1%)
Any positive serology (percent total tested)	7 (15.9%)	2 (10.5%)	9 (14.3%)

* Defined as a bat bite, bat scratch, or bat contact with unprotected skin.

**Table 2:** Risk factors for bat exposure among respondents in two communities in the Province Datem del Marañón of Perú, 2010

Subcategory	Exposed		Non-exposed		Total		*P* value	OR (95% CI)
	*n*	Percent	*n*	Percent	*n*	Percent		
Demographics (*N* = 92)								
Santa Marta	61	83.6	7	36.8	68	73.9	< 0.001*	8.71 (2.85–26.68)
Age less than or equal to 25 years†	41	56.2	5	26.3	46	50.0	0.038*	3.59 (1.17–11.01)
Male	43	58.9	8	42.1	51	55.4	0.207	1.97 (0.71–5.48)
Household characteristics								
More than five people living in household (*N* = 92)	52	71.2	8	42.1	60	65.2	0.029*	3.41 (1.20–9.65)
Own pets/livestock (*N* = 91)	63	87.5	17	89.5	80	87.9	1	0.82 (0.16–4.17)
Own dogs (*N* = 91)	24	33.3	11	57.9	35	38.5	0.065	0.36 (0.13–1.02)
Any pets/livestock bitten by bats (*N* = 79)	53	84.1	6	37.5	59	74.7	< 0.001*	8.83 (2.62–29.83)
Activities								
Lived in this house less than 1 year (*N* = 73)	7	13.0	8	42.1	15	20.5	0.017*	0.20 (0.06–0.69)
More than 5 years living/working with bats (*N* = 53)	12	32.4	2	12.5	14	26.4	0.183	3.36 (0.66–17.21)
Reported hunting bats (*N* = 70)	6	11.8	1	5.3	7	10.0	0.665	2.40 (0.27–21.37)
Reported agriculture (*N* = 70)	43	84.3	17	89.5	60	85.7	0.717	0.63 (0.12–3.29)
Reported using a mosquito net (*N* = 70)	37	72.5	14	73.7	51	72.9	1	0.94 (0.29–3.11)
Education (*N* = 76)								
Secondary or above	11	19.3	3	15.8	14	18.4	1	1.28 (0.32–5.16)
Knowledge (*N* = 70)								
Reported having basic or no rabies knowledge	51	100.0	19	100.0	70	100.0	NA‡	NA
Indicated animal bites as mechanism of transmission	11	21.6	5	26.3	16	22.9	0.752	0.77 (0.23–2.61)
Described rabies as severe	19	37.3	8	42.1	27	38.6	0.785	0.82 (0.28–2.39)
Identified bats as a rabies source	8	15.7	4	21.1	12	17.1	0.723	0.70 (0.18–2.65)
Identified dogs as a rabies source	16	31.4	9	47.4	25	35.7	0.266	0.51 (0.17–1.49)
If bitten by a bat (*N* = 70)								
Wash with soap and water	5	9.8	0	0.0	5	7.1	0.314	NA
Seek medical care	10	19.6	6	31.6	16	22.9	0.343	0.53 (0.16–1.74)
Do not know or do nothing	30	58.8	7	36.8	37	52.9	0.116	2.45 (0.83–7.26)
Other	6	11.8	6	31.6	12	17.1	0.074	0.29 (0.08–1.05)
If bitten by a rabid animal (*N* = 70)								
Wash with soap and water	2	3.9	0	0.0	2	2.9	1	NA
Seek medical care	16	31.4	10	52.6	26	37.1	0.163	0.41 (0.14–1.21)
Do not know or do nothing	33	64.7	7	36.8	40	57.1	0.056	3.14 (1.05–9.39)
Other	0	0.0	1	5.3	1	1.4	0.195	0.18 (0.09–0.34)
Vaccinated against rabies (*N* = 92)								
Post-exposure prophylaxis (bat exposure)	1	1.4	1	5.3	2	2.2	0.372	0.25 (0.015–4.19)
Pre-exposure prophylaxis (military service)	1	1.4	0	0.0	1	1.1	1	NA

* Statistically significant.

† Mean age of population.

‡ NA = not available.

**Table 3:** Indication of bat exposure and prior pre- or post-exposure prophylaxis history among seropositive survey respondents

Gender (age in years)	Location	RFFIT (IU/mL)	IFA		Bat exposure*	Bat bite	PreEP/PEP
			IgG	IgM			
Male (48)	Truenococha	0.4	1:128	–	Yes	No	No
Male (54)	Truenococha	ct	1:128	–	Yes	Yes	No
Male (34)	Santa Marta	0.6	–	–	Yes	Yes	No
Male (40)	Santa Marta	< 0.05	–	1:8	Yes	No	nd
Female (49)	Santa Marta	0.4	–	–	Yes	Yes	nd
Male (39)	Santa Marta	2.8	–	–	Yes	Yes	No
Male (49)	Santa Marta	0.4	–	–	Yes	Yes	No
Male (47)	Santa Marta	0.6	–	–	Yes	Yes	No
Female (27)	Santa Marta	0.1	1:8	–	Yes	Yes	PEP

* Bat exposure defined as a bite, scratch, or direct contact with unprotected skin.

ct = cytotoxic; IFA = indirect fluorescent antibody; IU = international unit; Ig = immunoglobulin; nd = not determined; PEP = post-exposure prophylaxis; PreEP = pre-exposure prophylaxis; RFFIT = rapid fluorescent focus inhibition test.

**Table 4:** Risk factors for exposure to rabies virus among respondents in two communities in the Province Datem del Marañón of Perú, 2010

Subcategory	Seropostive		Seronegative		Total		*P* value	OR (95% CI)
	*n*	Percent	*n*	Percent	*n*	Percent		
Demographics (*N* = 57)								
Age less than or equal to 29 years*	1	11.1	29	60.4	30	52.6	0.010†	0.08 (0.01–0.71)
Male	7	77.8	30	62.5	37	64.9	0.471	2.10 (0.39–11.23)
Santa Marta resident	7	77.8	31	64.6	38	66.7	0.703	1.92 (0.36–10.29)
Household characteristics								
More than five people living in household (*N* = 57)	6	66.7	28	58.3	34	59.6	0.726	1.43 (0.32–6.40)
Own pets/livestock (*N* = 56)	7	77.8	43	91.5	50	89.3	0.244	0.33 (0.05–2.13)
Own dogs (*N* = 56)	2	22.2	18	38.3	20	35.7	0.466	0.46 (0.09–2.46)
Any pets/livestock bitten by bats (*N* = 49)	6	85.7	29	69.0	35	71.4	0.656	2.69 (0.29–24.66)
Activities								
Lived in this house 1 year or less (*N* = 52)	1	12.5	11	25.0	12	23.1	0.663	0.43 (0.05–3.88)
More than 5 years living/working with bats (*N* = 41)	2	40.0	9	25.0	11	26.8	0.598	2.00 (0.29–13.94)
Reported hunting bats (*N* = 52)	2	25.0	4	9.1	6	11.5	0.227	3.33 (0.50–22.33)
Reported agriculture (*N* = 52)	8	100.0	39	88.6	47	90.4	1.000	NA‡
Reported using a mosquito net (*N* = 52)	1	12.5	14	31.8	15	28.8	0.412	0.31 (0.03–2.73)
Education (*N* = 52)								
Secondary or above	2	25.0	10	22.7	12	23.1	1.000	1.13 (0.20–6.51)
Knowledge (*N* = 52)								
Reported having basic or no rabies knowledge	8	100.0	44	100.0	52	100.0	NA	NA
Indicated animal bites as mechanism of transmission	3	37.5	11	25.0	14	26.9	0.666	1.80 (0.37–8.79)
Described rabies as severe	4	50.0	19	43.2	23	44.2	1.000	1.32 (0.29–5.95)
Identified bats as a rabies source	6	75.0	35	79.5	41	78.8	1.000	0.77 (0.13–4.48)
Identified dogs as a rabies source	3	37.5	19	43.2	22	42.3	1.000	0.79 (0.17–3.72)
If bitten by a bat (*N* = 52)								
Wash with soap and water	0	0.0	4	9.1	4	7.7	1.000	NA
Seek medical care	2	25.0	11	25.0	13	25.0	1.000	1.00 (0.18–5.70)
Do not know or do nothing	4	50.0	23	52.3	27	51.9	1.000	0.91 (0.20–4.12)
Other	2	25.0	6	13.6	8	15.4	0.593	2.11 (0.34–12.99)
If bitten by a rabid animal (*N* = 52)								
Wash with soap and water	0	0.0	2	4.5	2	3.8	1.000	NA
Seek medical care	3	37.5	19	43.2	22	42.3	1.000	0.79 (0.17–3.72)
Do not know or do nothing	5	62.5	23	52.3	28	53.8	0.711	1.52 (0.32–7.16)
Bat exposure								
Individuals with one or more bat exposures§ (*N* = 57)	9	100.0	36	75.0	45	78.9	0.180	NA
Bite (*N* = 57)	7	77.8	31	64.6	38	66.7	0.703	1.92 (0.36–10.29)
Bat bite more than one time per year (*N* =38)	2	28.6	10	32.3	12	31.6	1.000	0.84 (0.14–5.10)
Bite within the last 6 months (*N* = 38)	5	71.4	19	61.3	24	63.2	1.000	1.58 (0.26–9.48)
Contact with unprotected skin (*N* = 57)	5	55.6	16	33.3	21	36.8	0.266	2.50 (0.59–10.61)
Skin contact more than one time per year (*N* = 20)	1	20.0	6	40.0	7	35.0	0.613	0.38 (0.03–4.23)
Skin contact within the last 6 months (*N* = 21)	2	40.0	9	56.3	11	52.4	0.635	0.52 (0.07–4.00)
Scratch (*N* = 57)	0	0.0	3	6.3	3	5.3	1.000	NA
Inside a bat cave or refuge (*N* = 57)	1	11.1	7	14.6	8	14.0	1.000	0.73 (0.08–6.80)
Ate or cooked a bat as food (*N* = 57)	0	0.0	0	0.0	0	0.0	NA	NA
Vaccinated against rabies (*N* = 57)								
Post-exposure prophylaxis (bat exposure)	1	11.1	0	0.0	1	1.8	0.158	NA
Pre-exposure prophylaxis (military service)	0	0.0	1	2.1	1	1.8	1.000	NA

* Mean age of population.

† Statistically significant.

‡ NA = not applicable.

§ Defined as a bat bite, bat scratch, or bat contact with unprotected skin.
