# Supplementary material for: Evidence of Rabies Virus Exposure among Humans in the Peruvian Amazon
Source: Am J Trop Med Hyg. 2012 Aug 1;87(2):206–15. doi: 10.4269/ajtmh.2012.11-0689 (PMC3414554; doi:10.4269/ajtmh.2012.11-0689)
Supplement: Supplemental Questionnaire. [file SD1.pdf]

Supplemental Questionnaire

Knowledge, Attitudes, and Practices Questionnaire

A. HOUSEHOLD INFORMATION

1) Household ID number
2) GPS
3) Municipality
4) Community
5) Is there someone in the house that can respond to the interview?
   Yes
   No
6) If there is anyone in the house
6.1) Consent obtained?
   Yes
   No
6.2) If consent was NOT obtained
6.2.1) Reason for declining
   Not interested
   No time
   Fear of participating
   Not capable of consenting
   Language barrier
   Other
6.2.2) If the reason is "other"
6.2.2.1) Specify the reason for denying consent
6.3) If consent obtained
6.3.1) How many people live in this home?
6.3.2) How many are females living in this home?
6.3.3) How many children of ages 0–5 live in this house?:
6.3.4) How many children of ages 6–12 live in this house?:
6.3.5) How many children of ages 13–17 live in this house?:
6.3.6) About the house, which is the main material used to build the house?
   Brick
   Adobe
   Wood
   Canes
   Cement Block
   Cement/Concrete
   Other
6.3.7) If the house is made of another material
6.3.7.1) Specify the other housing material
6.3.8) Does the house have windows/doors that close and prevent bat entry? (Check all that apply)
   There are open windows
   Windows can close completely
   Doors can close completely
   There are windows or doors that close incompletely and allow entry of bats
   There are large openings in the walls for ventilation never closed
   No openings
   Other: _____________
   Don't know
   Declined to answer
6.3.9) Do you own animals as either pets or livestock?
   Yes
   No
   Don't know
   Declined to answer
6.3.10) If you own animals as either pets or livestock
6.3.10.1) Do you know or have you seen if your domestic animals (pets/cattle/pigs) have been bitten by bats?
   Yes
   No
6.3.10.2) If your domestic animals have been bitten by bats
6.3.10.2.1) Which of your animals have been bitten by bats? (Select all that apply?)
   Cows
   Goats
   Pigs
   Horses
   Dogs
   Cats
   Chicken
   Other
6.3.10.2.2) If the bitten animal is "Other"
6.3.10.2.2.1) Specify the animals that have been bitten by bats
6.3.10.3) Do you do anything to avoid your animals/pets being bitten by bats?
   Nothing
   Lights on where animals sleep
   Barriers (nets, close doors)
   Burn herbs
   Apply oil/chemicals to animals
   Hunt bats
   Blankets
   Garlic
   Other
6.3.10.4) If answered OTHER as something that is done to avoid your animals/pets being bitten by bats
6.3.10.4.1) Specify what other thing is done to avoid your animals/pets being bitten by bats
6.3.10.5) Are one or more of your animals vaccinated against rabies?
   Yes
   No
   Don't know
   Declined to answer
6.3.10.6) Have any of your animals been sick or died due to bats?
   Yes
   No
   Don't know
   Declined to answer
6.3.10.7) If any of your animals have been sick or died due to an illness that you believe may have been caused by bats?
6.3.10.7.1) What were their signs?
   Skin rash/discoloration/infection
   Unusual bleeding (e.g. from nose/mouth)
   Fever
   Cough
   Sneezing
   Runny nose
   Chest congestion
   Muscle pain
   Difficulty breathing
   Headache

Convulsions
Altered mental state (dementia)
Unconsciousness/coma
Muscle weakness/paralysis
Vomiting or diarrhea or stomach cramps
Miscarriage/stillbirth
Death
Multiple animals
Other
Don't know

6.3.10.7.2) If one of the signs is "Other"
6.3.10.7.2.1) Specify the other sign
6.3.10.7.3) Is the animal recovered from the symptoms?
Yes
No
Don't know
Decline to answer

6.3.10.7.4) What type of animal was it? (Mark all that apply)
Cows
Goats
Pigs
Horses
Dogs
Cats
Chicken
Other

7) Are there any dogs in your house?
Yes
No

7.1) If there are dogs in the house
7.1.1) How many dogs: ______
7.1.2) How many female dogs: ___________
7.1.3) How many male dogs: _____________
7.1.4) What is the age of the oldest dog in years?: ___
7.1.5) What is the age of the youngest dog?: ________
7.1.6) Are the dogs in your house vaccinated against rabies?:
Yes, all of them
Yes, but only some of them
No, none of them are vaccinated
Don't know
Decline to answer

7.1.6.1) If yes, when was the last vaccination:
Date: _________

7.1.7) Have any of the dogs in the house bitten anybody?
Yes
No
Don't know
Decline to answer

7.1.8) Have any of the dogs in the house bitten any of your other animals?
Yes
No
Don't know
Decline to answer

B. HOUSEHOLD RESPONDENT INFORMATION

1A) Sample ID
1B) Name: First name, First Family name, Second Family name
2) How old are you?
3) Gender
Male
Female

4) What is the last level of schooling you have completed?
None
Started primary
Completed primary
Started basic
Completed basic
Started secondary
Completed secondary
Started higher education
Completed higher education
Not sure
Declined to answer

5) How many years have you lived in this house?
Less than one year
One year
More than one year

6) If you have lived in this house more than one year
6.1) how many years
7) How many years of experience do you have working/living with or near bats?
None
5 or less
6–15
16–25
> 25
Don't know
Declined to answer

8) What activities do you engage in that regularly puts you in contact with bats? (check all that applies)
Bat hunting
Guano collection
Cattle farming
Quarry operator
Agriculture (sugar, coffee, etc.)
Nightwatchman
Other
None
Declined to answer

9) If the activity is Other
9.1) Specify
10) Have you been inside of a bat cave or bat refuge (trees, abandoned house, bridge, etc.)?
Yes
No
Don't know
Declined to answer

11) If you have been inside of a bat cave or refuge
11.1) How often do you enter bat caves or bat refuge?
Once per year or less
2–4 times per year
5 times or more per year
Don't know
Declined to answer

11.2) When was the last time you entered a bat cave or bat refuge?
< 1 month ago
1–6 months ago
6–12 months ago
More than 12 months ago
Don't know
Declined to answer

12) Have you ever touched a live bat with your skin uncovered?
Yes
No
Don't know
Declined to answer
13) If you have ever touched a live bat with your skin uncovered
13.1) How often do you touch bats?
Once per year or less
2–4 times per year
5 times or more per year
Don't know
Declined to answer
13.2) When was the last time you touched a bat with your skin uncovered?
< 1 month ago
1–6 months ago
6–12 months ago
More than 12 months ago
Don't know
Declined to answer
14) Have you ever been scratched by a bat, to your knowledge?
Yes
No
Don't know
Declined to answer
15) If has been scratched by a bat
15.1) How often are you scratched by bats?
Once per year or less
2–4 times per year
5 times or more per year
Don't know
Declined to answer
15.2) When was the last time you were scratched by a bat?
< 1 month ago
1–6 months ago
6–12 months ago
More than 12 months ago
Don't know
Declined to answer
16) Have you ever been bitten by a bat, to your knowledge?
Yes
No
Don't know
Declined to answer
17) If you have been bitten by a bat
17.1) How often are you bitten by bats?
Once per year or less 2–4 times per year
5 times or more per year
Don't know
Declined to answer
17.2) When was the last time you were bitten by a bat?
< 1 month ago
1–6 months ago
6–12 months ago
More than 12 months ago
Don't know
Declined to answer
18) Have you ever prepared a bat as food?
Yes
No
Don't know
Declined to answer
19) If you ever prepared a bat as food
19.1) How often do you prepare them for eating
Once per year or less
2–4 times per year
5 times or more per year
Don't know
Declined to answer
19.2) When was the last time you prepared one for eating?
< 1 month ago
1–6 months ago
6–12 months ago
More than 12 months ago
Don't know
Declined to answer
20) Have you ever eaten a bat?
Yes
No
Don't know
Declined to answer
21) If you have ever eaten a bat
21.1) How often do you eat bats?
Once per year or less
2–4 times per year
5 times or more per year
Don't know
Declined to answer
21.2) When was the last time you ate a bat?
< 1 month ago
1–6 months ago
6–12 months ago
More than 12 months ago
Don't know
Declined to answer
22) What kinds of bats do you most frequently observe or have had contact with?
Fruit-eating bats
Insect-eating bats
Vampire bats
Multiple types
Other
Don't know
Declined to answer
23) If the kind of bat is "Other"
23.1) Specify the other type of bat
24) Do you or your family do something to avoid bat bites in the house?
Nothing
Use mosquito net
Prevent entry of bat in the house
Increase number of cats
Increase the number of cattle/pigs to be bitten
Destroy bat refuges/kill bats
Pray
Declined to answer
Other
25) If answered OTHER as the type of action taken to avoid bites in the house
25.1) Specify what you and your family does to avoid bat bites in the house

26) How much do you know about rabies?
Little to none
Basic
Extensive
Declined to answer
27) How dangerous is rabies?
Very Severe
Mild or moderate
Don't know
Declined to answer
28) How do people get infected with rabies?
Animal bite
Animal scratch or lick
Touching an animal
Eating an animal
Other
Don't know
Declined to answer
29) If the way people are infected with rabies is "Other"
29.1) Specify the way people are infected by rabies
30) What animals can be infected with rabies? (check all that apply)
Bats
Dogs
Cats
Horses
Livestock
Wild mammals (not bats)
Other
Don't know
Declined to answer
31) If the animals are potentially infected with rabies are "Other"
31.1) Specify which other animals could be infected with rabies
32) What would you do if you were bitten or scratched by a bat?
Nothing
Wash wound with soap and water
Call a doctor for advice
Call or visit a traditional healer
Seek medical care at a hospital, clinic or health post
Seek rabies PEP
Have bat tested for rabies (or other diseases)
Other
Don't know
Declined to answer
33) If the action that you would take is Other
33.1) Specify the other action that would be taken
34) Do you think there is any time of the year in which bats attack more animals or people?
No, it is the same all year round
Yes, rainy season (April–October)
Yes, dry season (November–April)
Don't know
Declined to answer
35) If someone has been bitten by an animal potentially infected with rabies what should that person do? (Check all that apply)
Nothing
Wash wound with soap and water
Call a doctor for advice
Call or visit a traditional healer
Seek medical care at a hospital or clinic
Seek rabies post-exposure prophylaxis
Check animal's vaccination history
Observe animal for a period of time to see if it becomes rabid
Have animal tested for rabies
Kill animal
Other
Don't know
Declined to answer
36) If the action is "Other"
36.1) Specify the other action that should be done if someone has been bitten by an animal that might be infected by rabies
37) Have you ever been vaccinated against rabies?
Yes
No
Don't know
Declined to answer
38) If you have ever been vaccinated against rabies
38.1) What was the reason you were vaccinated against rabies?
Post-exposure prophylaxis
Pre-exposure prophylaxis
Have received PreEP and PEP
Don't know
Declined to answer
38.2) If you have received rabies vaccination after being bitten or scratched by an animal bite, what animal or animals were responsible for the incident? (check all that apply)
Bats
Dogs
Cats
Horses
Livestock
Wild mammals (not bats)
Others
Don't know
Declined to answer
Did not receive PEP
38.3) If received a vaccination after being bitten by an OTHER animal
38.3.1) Specify the other animal that bit you
39) Are you aware if there are any other diseases that humans can get from bats?
Yes
No
Don't know
Declined to answer
40) Have you or anyone you know ever experienced an illness that you believe may have been caused by bats or being in a bat cave?
Yes
No
Don't know
Declined to answer
41) If you or anyone you know ever experienced an illness that you believe may have been caused by bats or being in a bat cave
41.1) What were the symptoms? (Check all that apply)
Skin rash/discoloration/ infection
Unusual bleeding (e.g. from nose/mouth)
Fever
Cough

Sneezing
Runny nose
Chest congestion
Muscle pain
Difficulty breathing
Headache
Convulsions
Altered mental state (dementia)
Unconsciousness/coma
Muscle weakness/paralysis
Vomiting or diarrhea or stomach cramps
Miscarriage/stillbirth
Death
Multiple persons
Other
Don't know
Declined to answer

41.2) If the symptoms is "Other"
41.2.1) Specify the other symptom
41.2.2) Are you or the person you know that presented symptoms caused by a bat recovered?
Yes
No
Don't know
Decline to answer

42) We would like to take a sample of your blood. Will you allow us to take a sample?
Yes
No

42.2) Was blood sampled obtained?
□ Yes
□ No

42.3) IF blood sample was not obtained, why not?
□ Did not consent for blood
□ Was not able to get blood
□ Other: _______________

43) Has anyone from your family or living here been in contact, bitten, scratched, eaten, or touched a bat?*
Yes
No

*Additional persons identified with exposures were surveyed using the questions in Section B. HOUSEHOLD RESPONDENT INFORMATION
